# Role of Transverse Shear Modulus in the Performance of Corrugated Materials

**DOI:** 10.3390/ma13173791

**Published:** 2020-08-27

**Authors:** Tomasz Garbowski, Tomasz Gajewski, Jakub Krzysztof Grabski

**Affiliations:** 1 Institute of Structural Analysis, Poznan University of Technology, Piotrowo Street 5, 60-965 Poznań, Poland; tomasz.garbowski@put.poznan.pl; 2 Institute of Applied Mechanics, Poznan University of Technology, Jana Pawła II Street 24, 60-965 Poznań, Poland; jakub.grabski@put.poznan.pl

**Keywords:** corrugated board, transverse shear modulus, laboratory tests, box performance, torsion

## Abstract

In a description of materials for orthotropic panels with a soft and/or corrugated core, it is important to correctly determine all constitutive parameters. In laboratory practice, the determination of transverse shear modulus is often overlooked. This paper presents a method for determining this property based on a plate torsion test and a correctly formulated analytical description. It has been proved that the transverse shear effect in some cases cannot be omitted because it significantly influences the mechanical behavior of corrugated board. The method of transverse shear modeling used so far can be modified to eliminate dimensionless, physically unjustified coefficient and replace them with coefficients that have a physical basis. It is shown here that such modification leads to results with lower error. The effective modeling of transverse shear effects enables a more conscious design of corrugated board structures, where the final goal is to obtain packaging with high strength and durability but low material consumption.

## 1. Introduction

Corrugated materials and structures have been used in mechanical engineering for many years, their application may have various characters from webs for I-beam [1] through very unique natural lattice sandwich structures [2] to a more conventional function of supporting structures in the corrugated board packaging industry.

In recent years, corrugated board packaging has gained popularity in transport and logistics. The main reason for the increased interest in these products is the ever-growing e-commerce market. Internet sales naturally stimulate the market for transport services, which leads to increased demand for transport boxes. The economic and ecological aspects of this constantly increasing demand for corrugated packaging cannot be ignored. For effective recycling of wastepaper, many large production and trade companies use corrugated cardboard as a safe and environmentally friendly material for packaging their products. Effective recycling is one of the tools of the currently implemented sustainable development policies. In response to growing market requirements, the paper packaging industry constantly raises the standards of its packaging, not only as it relates to recycling but also for more effective use of materials (paperboard and corrugated cardboard).

Trading companies are interested in ensuring the quality of their products, which should be well-protected in attractive, recognizable, and branded packaging. However, companies that produce corrugated packaging aim for lightweight products to reduce production costs. This is why packaging made of corrugated board must often be attractive and light, but also stabile and resistant. Additionally, production cost should be low (through the use of recycled paper and/or optimal packaging) and good printability should be ensured (through a special layer of outer paper).

Unfortunately, printing on the cardboard reduces the strength of the packaging. If a digital printing technique is used, the degree of cardboard damage is minimal. However, when flexographic or offset techniques are used, the damage to the printed material is greater. Because digital printing technology today accounts for a minority of the printing in the global market, the majority of printed packaging is to a greater or lesser extent weakened by printing technology. This phenomenon occurs mainly due to pressure during printing, which causes damage to the corrugated layers, and in turn leads to a reduction in the bending stiffness and torsion stiffness of the corrugated board. As a consequence, the degraded rigidity directly reduces the strength of the packaging for both static and dynamic loads.

Therefore, manufacturers of corrugated board packaging should be able to determine to what extent the printing decreases the load capacity of the designed box. One way to do this is to estimate the load capacity of the packaging using analytical formulas, e.g., the popular McKee formula [3]. Unfortunately, the analytical formulas do not include all the parameters that degrade during printing, namely the thickness, the transverse shear stiffness in the machine direction (MD), and the transverse shear stiffness in the cross direction (CD). Another possibility is to check the decrease in load capacity of the printed packaging by using the box compression test (BCT). Unfortunately, if the load capacity is checked after box production has started (a posteriori), and the decrease is found to be too large, it is often too late to correct the construction or change the cardboard quality. In this case, either production must stop and resume with a new design and production setup, or the risk of returns and costly complaints must be accepted.

In the literature, one can find some modifications of the McKee formula. Review of different formulas for box compression analysis and an improved approach were presented by Urbanik and Frank [4]. Recently, Coffin examined the McKee formula modified by Urbanik and Frank for BCT using the box data coming from the original McKee’s work and the data of Batelka and Smith [5]. Schaffrath et al. examined the influence of asymmetric properties of boxes on robustness of the McKee formula for prediction of box strength [6]. However, torsional test and transverse stiffness of boxes are considered very rarely in the literature.

An alternative method that has recently become more popular is load capacity prediction based on numerical methods, which requires the definition of cardboard properties. These properties can be obtained through adequate laboratory testing [7,8], and include transverse shear stiffness in the MD ($G_{13}$ modulus) and the CD ($G_{23}$ modulus). Determining these parameters, although not complicated, is unfortunately rarely done. Knowing these parameters, especially for material that is converted (after printing, etc.), allows for an assessment of the extent to which the material (corrugated cardboard) has been damaged [9] and, consequently, the extent to which the strength of the final packaging has been reduced.

**Figure 1:**
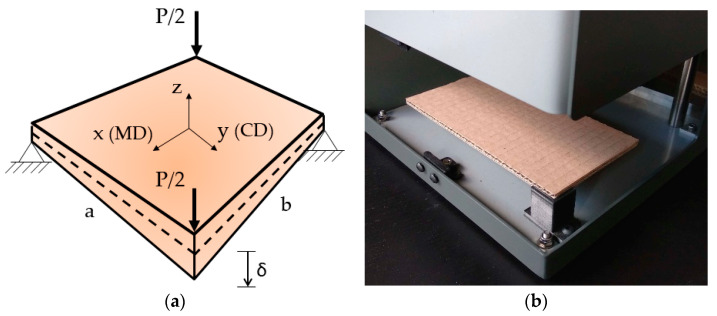
(**a**) Scheme of the static plate torsion test; (**b**) visualization of the sample in the machine during test.

In this work, a method for calculating the transverse stiffness of corrugated cardboard based on modified plate torsion tests (Figure 1) and analytical formulas is presented. There have been many attempts in the literature to determine and/or describe these properties [10,11,12,13,14,15,16]. One of the classical studies on the subject is the work of Reissner [17]. The approach presented in 1980 in his work regarding the prediction of torsional stiffness of beams and plates has become fundamental. Whitney, in his work from 1987 [18], gathered knowledge about the shear deformation in laminated anisotropic plates. He concluded that the classical laminate plate theory (CLPT) was not sufficient to model the behavior of laminated plates. More recent work by Popil et al. [19] deals with transverse shear measurement. The authors considered corrugated boards, and twisting stiffness in two directions; MD and CD, was introduced with the variables $k_{MD}$ and $k_{CD}$, respectively. For $k_{MD},k_{CD}$, the transverse shear modulus was considered in the sample dominant direction only. We prove that both transverse shear moduli are “active”, but the one in the non-dominant direction plays a minor role, however they should be included.

Recently, the Carlsson group made an important contribution to this research area, namely in studies by Avilés et al. [20,21,22] and Hernandez-Perez et al. [23,24]. In their work from 2009, Avilés et al. [20] compare the results from CLPT with the finite element method (FEM) for sandwich (soft-core) plates. The conclusion was that the classical theory was not sufficient for modeling a twist in such structures. In the work from 2011, Avilés et al. [21] focused on experiments on the torsion and shear properties of sandwich panels and laminated composites. They made the first reference to a preliminary shear-corrected model, which was explicitly stated in the paper by Avilés et al. from 2012 [22]. In addition, in 2012, Hernandez-Perez et al. [23] studied the twist test in sandwich plates, comparing results based on first-order shear deformation (FOSD) theory and finite element computations. The applicability of the theory was evaluated as acceptable, but with some limitations. However, mainly soft-core structures were analyzed. In their 2014 study [24], Hernandez-Perez et al. redirected their interest to the corrugated boards of single- and double-walled structures. It should be highlighted that for all numerical simulations of corrugated boards, including FEM calculations, the transverse shear moduli should be determined in an appropriate way [25,26]. In this study, experimental, structural FEM, homogenized FEM, CLPT, and FOSD results were compared. The FOSD and FEM predictions showed good performance compared with the experimental results. The CLPT results were highly overestimated.

In our study, the modification by Avilés et al. from 2012 [22] is developed. It may be classified as one of the FOSD method and serve as an alternative to the FOSD approach presented by Hernandez-Perez et al. in their 2014 study [24]. Another example of FOSD was presented by Nguyen et al. in 2019 [27] for advanced composite plates such as functionally graded plates, where no shear correction factor was used. In the literature, also the alternatives to FOSD may be found, for instance in the work of Anish et al. [28], the so-called improved shear deformation theory was presented, in which a cubic variation of the thickness coordinate in the displacement field is employed.

## 2. Materials and Methods

### 2.1. Modified Analytical Approach

In order to correctly identify the properties of cardboard as a construction material, it is necessary to perform a series of laboratory tests. The following properties describe the constitutive constants of corrugated board in the elastic range (see Figure 1):

- $D_{11}$—bending stiffness in the MD,
- $D_{22}$—bending stiffness in the CD,
- $D_{66}$—twisting bending stiffness,
- $A_{11}$—compression stiffness in the MD,
- $A_{22}$—compression stiffness in the CD,
- $A_{33}$—compression stiffness in the z direction (out of plane),
- $A_{44}$—transverse shear stiffness in the 1–3 (x–z) plane,
- $A_{55}$—transverse shear stiffness in the 2–3 (y–z) plane.

These quantities occur in the constitutive equations that describe shell elements:

(1) $$A=\left[\begin{array}{ccc} A_{11} & A_{12} & 0\\ A_{12} & A_{22} & 0\\ 0 & 0 & A_{33} \end{array}\right];\;\;\;B=\left[\begin{array}{ccc} D_{11} & D_{12} & 0\\ D_{12} & D_{22} & 0\\ 0 & 0 & D_{66} \end{array}\right];\;\;\;\;\;\Gamma =\left[\begin{array}{cc} A_{44} & 0\\ 0 & A_{55} \end{array}\right],$$

and can be represented by the following formulas:

(2) $$D_{11}=E_{1}\cdot \frac{t^{3}}{12w};\;\;D_{22}=E_{2}\cdot \frac{t^{3}}{12w};\;\;D_{12}=D_{22}\nu_{12};\;w=1-\nu_{12}\nu_{21},$$

(3) $$D_{66}=G_{12}\cdot \frac{t^{3}}{12},$$

(4) $$A_{11}=\;E_{1}t;\;\;A_{22}=E_{2}t\;;\;\;A_{12}=A_{22}\nu_{12},$$

(5) $$A_{44}=\frac{5}{6}G_{13}t;A_{55}=\frac{5}{6}G_{23}t,$$

where:

- t—effective cardboard thickness,
- $E_{1}$—effective stiffness modulus in the MD,
- $E_{2}$—effective stiffness modulus in the CD,
- $\nu_{12}=0.293\sqrt{E_{1}/E_{2}}$—effective Poisson’s ratio in the 1–2 (x–y) plane [29],
- $v_{21}=v_{12}E_{2}/E_{1}$—effective Poisson’s ratio in the 1–2 (x–y) plane,
- $G_{12}$—effective shear modulus in 1–2 (x–y) plane,
- $G_{13}$—effective transverse shear modulus in the 1–3 (x–z) plane,
- $G_{23}$—effective transverse shear modulus in the 2–3 (y–z) plane.

A plate torsion test may be used to determine the shear stiffness of a particular corrugated cardboard sample. The compliance of sample C from the plate torsion test takes the following form:

(6) $$C=\frac{\delta }{P},$$

where δ is the sample displacement (in this study δ=1 mm) and P is the reaction force measured by a torsion testing device [7]. The compliance of the sample proposed in the work of Avilés et al. [22], compared to the classical laminate theory [17,18], was an extension of the traditional approach by adding a transverse shear member, namely:

(7) $$C=C_{c}+C_{s},$$

where $C_{c}$ is the compliance from classical laminate theory, and $C_{s}$ is the contribution due to the transverse shear. According to classical laminate theory:

(8) $$C_{c}=\frac{ab}{16D_{66}},$$

where a and b are the dimensions of the corrugated cardboard sample, and $D_{66}$ is the torsion stiffness. Parameter $D_{66}$ is computed using Equation (3). Based on [22], the contribution due to the transverse shear $C_{s}$ is expressed by the following:

(9) $$C_{s}=\frac{c_{1}}{\sqrt{A_{44}\cdot A_{55}}},$$

where $c_{1}$ is the nondimensional constant, $A_{44}$ is the transverse shear stiffness in the 1–3 (x–z) plane, and $A_{55}$ is the transverse shear stiffness in the 2–3 (y–z) plane. $A_{44}$ and $A_{55}$ are computed from Equation (5).

The dependence of variable $C_{s}$ on the transverse shear modulus $G_{13}$ and $G_{23}$ in Equations (9) and (5) seems to be a reasonable assumption. However, the approach is still not practical to effectively determine the transverse shear contribution to the performance of the corrugated cardboard sample. In the approach proposed in [22], the use of Equation (9) requires determination of the coefficient $c_{1}$, which, despite the analyses presented in the cited work, seems to be a non-trivial task.

The nondimensional constant $c_{1}$ can be determined by rearranging the equations above. The substitution of Equations (6), (8) and (9) into Equation (7) leads to the following form:

(10) $$\frac{\delta }{P}=\frac{ab}{16D_{66}}+\frac{c_{1}}{\sqrt{A_{44}\cdot A_{55}}}.$$

Assuming δ=1 mm, simple mathematical operations of Equations (3), (5), and (10) enable the following form of $c_{1}$:

(11) $$c_{1}=\left(\frac{1}{P}-\frac{3ab}{4G_{12}t^{3}}\right)\cdot \frac{5}{6}t\sqrt{G_{13}\cdot G_{23}}.$$

Knowing the reference value of force P (Figure 1a) derived from numerical simulations, one can determine the range of application of the coefficient $c_{1}$ for different (assumed) values of $a,b,t,\;G_{12},G_{13},$ and $G_{23}$.

### 2.2. Computational Model

In this research study, the reference values of forces P were obtained using the FEM by performing linear static analyses. Calculations were conducted in the Abaqus FEM commercial program. A (homogenized) model of shell theory with three-node triangular elements was used. Assuming a plane stress state in the shell, an orthotropic material model was used. The in-plane relation of the stress vector σ and the strain vector ε can be described by the following relationship:

(12) $$\left\{\begin{array}{c} \varepsilon_{1}\\ \varepsilon_{2}\\ \gamma_{12} \end{array}\right\}=\left[\begin{array}{ccc} \frac{1}{E_{1}} & -\frac{\vartheta_{21}}{E_{2}} & 0\\ -\frac{\vartheta_{12}}{E_{1}} & \frac{1}{E_{2}} & 0\\ 0 & 0 & \frac{1}{G_{12}} \end{array}\right]\left\{\begin{array}{c} \sigma_{11}\\ \sigma_{22}\\ \tau_{12} \end{array}\right\},$$

where $E_{1}$, $E_{2}$, $\vartheta_{12}$, and $G_{12}$ are the effective longitudinal elasticity modules in direction 1 (MD) and direction 2 (CD), the effective Poisson’s ratio, and the effective in-plane shear modulus, respectively.

The boundary conditions of the sample were assumed to emulate a support in the plate torsion test. The translational degrees of freedom were blocked at the opposite corner nodes of the plate (Figure 1a). A kinematic constraint was introduced in the other corners of the plate to simulate displacement application during testing. It is worth noting that P was the overall force applied during the test, thus the reaction forces at the corners from the simulations were equal to half the force P.

## 3. Results

Depending on the particular sample of corrugated cardboard, a transverse shear contribution may have an important effect on material performance. In this paper, using the formulas presented in Section 2.1 and the model described in Section 2.2, the contribution of the transverse shear to total compliance C was verified (here C=1/P). In other words, the ratio $C_{s}$ to C was computed.

**Figure 2:**
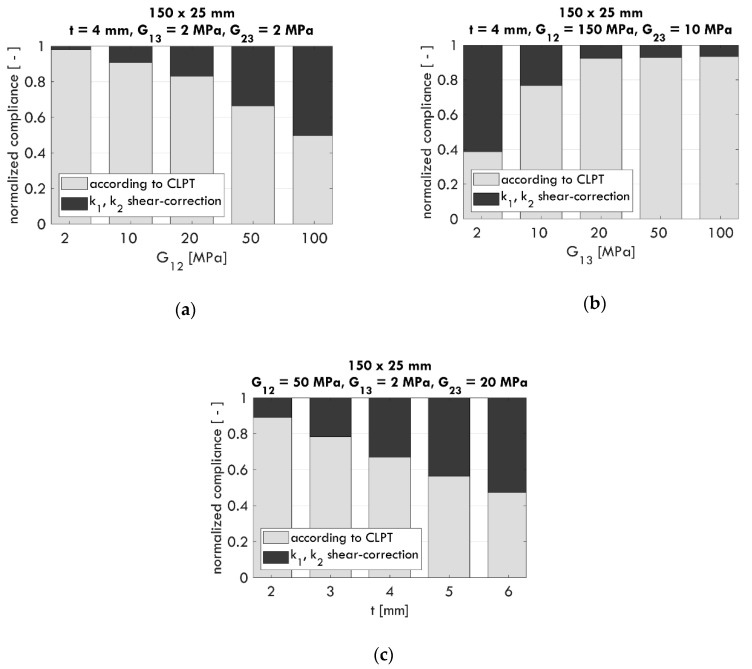
Examples of significant influence of transverse shear on sample compliance for selected values of G_12_, G_13_, G_23_, and t with respect to changing values of G_12_ (**a**), G_13_ (**b**), and t (**c**).

In Figure 2, the bar charts for compliance 1/P normalized to 1 are shown, where for the example combinations of $G_{12},\;G_{13},\;G_{23},$ and t, the ratios $C_{c}/C$ (gray bars) and $C_{s}/C$ (black bars) were calculated. The assumed dimensions of the sample were 150×25 mm. The high ratio of $C_{s}/C$ shows how significant the impact of the transverse shear was on the compliance for a given cardboard sample. Depending on the adopted material parameters and sample thickness, this value may even reach several dozen percent, e.g., 37% for $G_{12}$ = 100 MPa (Figure 2a), 61% for $G_{13}$ = 2 MPa (Figure 2b), and 53% for t = 6 mm (Figure 2c). Therefore, it can be concluded that there are cases in which it is necessary to account for the transverse shear correction $C_{s}$ in relation to the use of (pure) classical theory, i.e., Equation (8).

**Figure 3:**
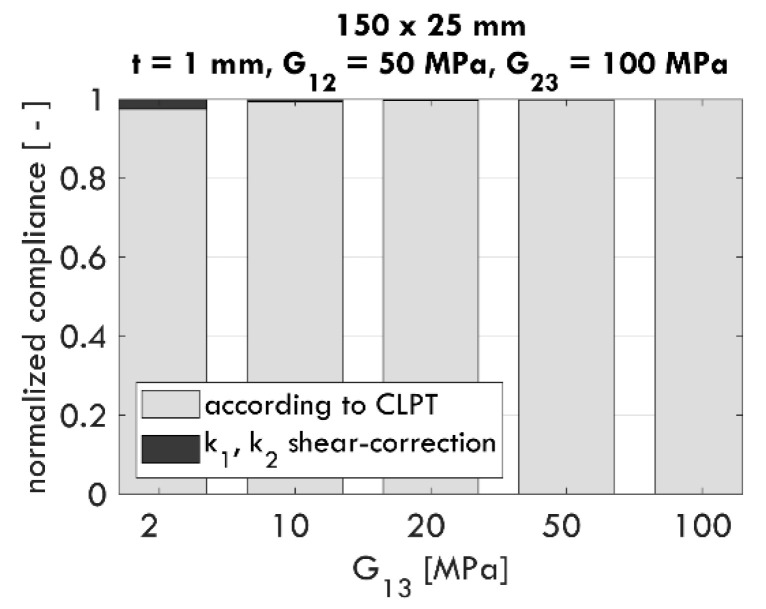
Example of a negligible influence of transverse shear on sample compliance for selected values of $G_{12},\;G_{23}$, and t with respect to the change in $G_{13}$.

In some cases, particularly for thin cardboard, the effect of transverse shear is negligible. This phenomenon is illustrated in Figure 3, where for t = 1 mm and changing values of $G_{13}$, the influence of $C_{s}$ on C is usually less than 2.5%. In such cases, applying only classical theory ($C_{c}$), excluding $C_{s}$, is accurate enough.

The shear correction proposed in [22] and shown as Equation (9) introduces the questions: (i) what should be the value of the dimensionless coefficient $c_{1}$ to obtain the correct compliance value, and (ii) what is the influence on $c_{1}$ if the material parameters, sample dimensions, or thickness change? In order to answer these questions, the sensitivity of $c_{1}$ was systematically tested, and selected computational results are presented later in this work.

**Figure 4:**
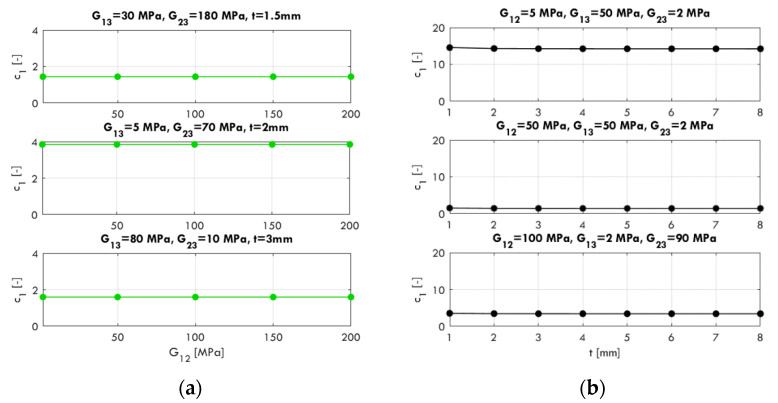
Influence of $G_{12}$ (**a**) and effective thickness t (**b**) on the value of the $c_{1}$ parameter.

In Figure 4, the calculated values of $c_{1}$ according to Equation (11) for certain combinations of material parameters ($G_{12},\;G_{13},$ and $G_{23}$), thickness t, and sample dimensions are shown. The following sample dimensions are adopted: 25×150 mm, 150×75 mm, and 75×75 mm. Regardless of the $G_{12}$ value (Figure 4a) and sample thickness t (Figure 4b), the values of the computed coefficient $c_{1}$ are constant. Based on the results, it can be concluded that $c_{1}$ does not depend on $G_{12}$ and t.

**Figure 5:**
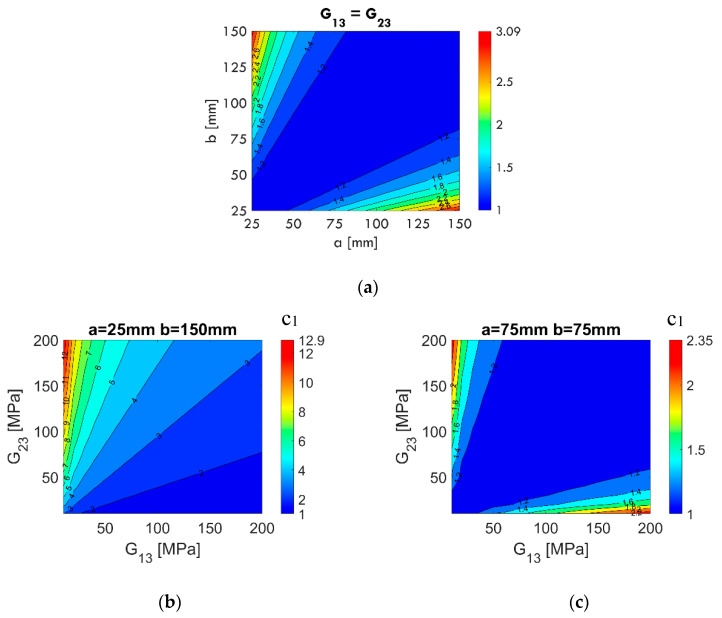
Influence of the relation a/b (**a**) and $G_{13}/G_{23}$ for a sample of 25 × 150 mm (**b**), and a sample of 75 × 75 mm (**c**), on the value of the $c_{1}$ parameter.

In the next step, two aspects of $c_{1}$ are analyzed, i.e., the impact of sample aspect ratio for constant and equal transverse shear moduli ($G_{13}=G_{23}$) and the relation between transverse shear moduli $G_{13},\;G_{23}$ (the selected sample sizes a×b were 25×150 mm and 75×75 mm). In Figure 5a, the influence of dimensions a and b is shown. The values of $c_{1}$ reach a maximum of about 3.09 for a longitudinal sample with a dimension aspect ratio of 1:6 (25×150 mm), i.e., the transverse shear correction due to the sample geometry should be significant. For square samples, e.g., 50×50 mm, 125×125 mm, the factor $c_{1}$ should be 1 (no correction). The contour plot of $c_{1}$ values is symmetrical in relation to the main diagonal, i.e., the transverse shear correction for the 25×150 mm and 150×25 mm samples is the same, assuming $G_{13}=G_{23}$. On the contrary, in Figure 5b, the effect of cross shear modulus relationships for a rectangular sample (a = 25 mm, b = 150 mm) is shown. The variable $c_{1}$ reaches a value of around 12.9, a very significant effect of transverse shear correction due to the relationship of $G_{13}/G_{23}$ ($G_{13}$ = 10 MPa and $G_{23}$ = 200 MPa). In Figure 5c, the same relationship for a square sample (a= 75 mm, a= 75 mm) is shown. The maximum value of $c_{1}$ in this case is approximately 2.35. On this contour plot, one can see a symmetry with respect to the main diagonal, similar to the case shown in Figure 5a.

Based on the systematic calculations of $c_{1}$, a modification of Equation (9) was proposed. Two physically justified coefficients, $k_{1}$ and $k_{2}$, were introduced instead of coefficient $c_{1}$. Thus, the correction due to shear takes the following form:

(13) $$C_{s}=\frac{k_{1}\cdot k_{2}}{\sqrt{A_{44}\cdot A_{55}}},$$

(14) $$\begin{array}{cc} k_{1}=\frac{2}{5}\left(\frac{b}{a}+\frac{7}{5}\right), & b>a \end{array},$$

(15) $$\begin{array}{cc} k_{2}=\frac{3}{5}{\left(\frac{a}{b}\cdot \frac{G_{23}}{G_{13}}\right)}^{\frac{2}{5}}, & G_{23}>G_{13} \end{array},$$

where $k_{1}$ is a factor that accounts for the proportion of sample dimensions, and $k_{2}$ is a factor that accounts for the ratio $G_{23}\;/G_{13}$. Both dimensionless coefficients were proposed based on an approximation of the systematic computations made.

In the next step, a numerical verification of Equations (13)–(15) that takes into account the transverse shear effect was carried out. Intervals for transverse shear moduli $G_{13},\;G_{23}$ from 2 to 300 MPa (6 discrete values for $G_{13}$ and $G_{23}$, namely, 2, 20, 50, 100, 200, and 300 MPa) and sample dimensions from 25 to 150 mm (6 discrete values for a and b, namely, 25, 50, 75, 100, 125, and 150 mm) were assumed. Using all possible combinations for the values of $G_{13},\;G_{23}$, a and b values, 1296 computational cases (6^4^) were obtained. Based on Equation (6) (assuming that δ = 1) and using Equations (7), (8), and (13)–(15), the values of P were calculated. For the same input data, the reference values of forces $P_{ref}$ were obtained using the FEM (see Section 2.2, in which the computational model used was described).

**Figure 6:**
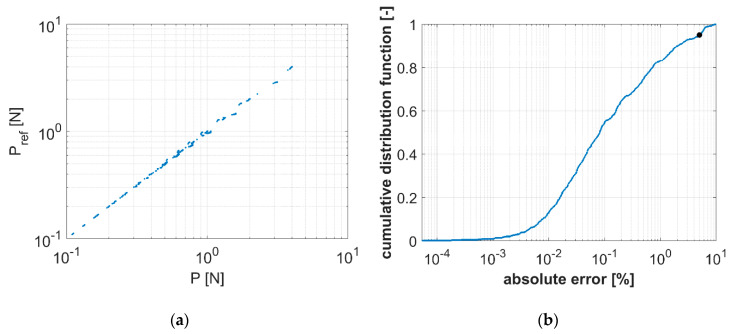
Solution error for P from the modified analytical approach, and $P_{ref}$, the reference values derived from the FEM: (**a**) logarithmic regression plot of raw data and (**b**) cumulative distribution function plot of the data.

The differences between P and $P_{ref}$ are graphically presented in Figure 6. Figure 6a shows raw data, where on the vertical axis the reference values of the force *P_ref_* are presented, while on the horizontal axis, its counterparts computed by our proposed approach are demonstrated. In Figure 6a, almost perfect alignment of the magnitudes can be observed. Figure 6b presents a histogram of the data. In may be observed that the error is less than 5% for about 95% of all cases (see black dot). The average overall error for the solutions of the 1296 cases is 0.11%. The error is 10.1% in only one case; in all other cases, it is less than 10%. The largest errors (greater than 5%) were obtained in cases where G13 and G23 reached the limit values, e.g., G13 = 2 MPa and at the same time G23 = 300 MPa. Such values of these parameters are usually non-physical, which would mean that the material has very high shear stiffness in one direction and virtually no shear stiffness in other, perpendicular direction. All raw data used to generate Figure 6 can be found in Supplementary materials.

For the classical solution (using $c_{1}$ as an a priori constant), the largest error in estimating P according to [22] for $c_{1}=1$ is equal to 35% (average error 2.5%), and for $c_{1}=2$, the largest error is equal to 32% (average error 1.2%).

## 4. Discussion

In this paper, the effect of the contribution of transverse shear on the results of torsion tests of corrugated cardboard plates was analyzed. It was proved by using numerical and analytical approaches that in some configurations, not including the transverse shear, may lead to results with a significant error. Therefore, it is proposed to modify the analytical form available in the literature to account for a transverse shear effect. Based on the calculations carried out, by eliminating the dimensionless physically unjustified coefficients, the mechanical effects observed were introduced into the formulas, and those with no significant influence were ignored. The effectiveness of the newly proposed method was proved by comparing the results with the FEM calculations.

We conclude that a measurement of torsional stiffness in certain configurations of sample size ratio and for small (degraded) values of transverse stiffness moduli can be effectively used to determine the transverse shear stiffness of corrugated cardboard. This is especially important when the corrugated layers of cardboard are damaged due to traditional printing methods, which directly reduce the load capacity of the packaging. The load capacity of the packaging is largely influenced by the edge crushing resistance of cardboard (ECT), and its bending stiffness. The last, though not least important property that affects the load capacity of corrugated board packaging is the transverse shear stiffness. Its influence is crucial, especially when the cardboard is crushed during printing, lamination, etc. The correct determination of this property is therefore very important for a reliable estimation of the strength of cardboard packaging after processing.

## Supplementary Materials

The following are available online at https://www.mdpi.com/1996-1944/13/17/3791/s1. Figure 6 was presented as CSV file, in which 1st column are the numbers of the case analyzed, 2nd and 3rd columns are a and b dimensions of the sample, 4th and 5th columns are G13 and G23 assumed for particular case, 6th column are the values obtained from the modified analytical approach, 7th column are the reference values derived from the FEM and 8th column are the error values between 5th and 6th columns.

## References

[1] Górecki M., Śledziewski K. (2020). Experimental investigation of impact concrete slab on the bending behavior of composite bridge girders with sinusoidal steel web. Materials.

[2] Li S., Feng Y., Wang M., Hu Y. (2018). Mechanical behavior of natural fiber-based bi-directional corrugated lattice sandwich structure. Materials.

[3] McKee R.C., Gander J.W., Wachuta J.R. (1963). Compression strength formula for orrugated boxes. Paperboard Packag..

[4] Urbanik T.J., Frank B. (2006). Box compression analysis of world-wide data spanning 46 years. Wood Fiber Sci..

[5] Coffin C.W. (2015). Some observations towards improved predictive models for box compression strength. TAPPI J..

[6] Schaffrath H.J., Reichenbach F., Schabel S. (2018). Prediction of box failure from paper data for asymmetric corrugated board. TAPPI J..

[7] FEMat Systems. http://www.fematsystems.pl/en/systems/sst/.

[8] Garbowski T., Garbowska L. (2018). Computer aided estimation of corrugated board box compression strength. Part 3. Laboratory-numerical procedure for an identification of elastic properties of corrugated board. Pol. Pap. Rev..

[9] Garbowski T., Czelusta I., Graczyk Ł. (2018). Computer aided estimation of corrugated board box compression strength. Part 1. The influence of flute crash on basic properties of corrugated board. Pol. Pap. Rev..

[10] Cohen G.A. (1978). Transverse shear stiffness of laminated anisotropic shells. Comput. Method Appl. Mech. Eng..

[11] Nordstrand T., Carlsson L.A., Allen H.G. (1994). Transverse shear stiffness of structural core sandwich. Comp. Struct..

[12] Nordstrand T.M. (1995). Parametric study of the post-buckling strength of structural core sandwich panels. Comp. Struct..

[13] Shi G., Tong P. (1995). Equivalent transverse shear stiffness of honeycomb cores. Int. J. Solids Struct..

[14] Altenbach H. (2000). An alternative determination of transverse shear stiffnesses for sandwich and laminated plates. Int. J. Solids Struct..

[15] Carlsson L.A. (2001). On the elastic stiffnesses of corrugated core sandwich. J. Sandw. Struct. Mater..

[16] Nordstrand T. (2004). On buckling loads for edge-loaded orthotropic plates including transverse shear. Comp. Struct..

[17] Reissner E. (1980). On torsion and transverse flexure of orthotropic elastic plates. J. Appl. Mech..

[18] Whitney J.M. (1987). Structural Analysis of Laminated Anisotropic Plates.

[19] Popil R.E., Coffin D.W., Habeger C.C. (2008). Transverse shear measurement for corrugated board and its significance. Appita J..

[20] Avilés F., Carlsson L.A., Browning G., Millay K. (2009). Investigation of the sandwich plate twist test. Exp. Mech..

[21] Avilés F., Couoh-Solis F., Carlsson L.A., Hernández-Pérez A., May-Pat A. (2011). Experimental determination of torsion and shear properties of sandwich panels and laminated composites by the plate twist test. Comp. Struct..

[22] Avilés F., Carlsson L.A., May-Pat A. (2012). A shear-corrected formulation of the sandwich twist specimen. Exp. Mech..

[23] Hernandez-Perez A., Aviles F., Carlsson L.A. (2012). First-order shear deformation analysis of the sandwich plate twist specimen. J. Sandw. Struct. Mater..

[24] Hernández-Pérez A., Hägglund R., Carlsson L.A., Avilés F. (2014). Analysis of twist stiffness of single and double-wall corrugated boards. Comp. Struct..

[25] Garbowski T., Jarmuszczak M. (2014). Numerical strength estimate of corrugated board packages. Part 1. Theoretical assumptions in numerical modeling of paperboard packages. Pol. Pap. Rev..

[26] Garbowski T., Jarmuszczak M. (2014). Numerical Strength Estimate of Corrugated Board Packages. Part 2. Experimental tests and numerical analysis of paperboard packages. Pol. Pap. Rev..

[27] Nguyen H.N., Hong T.T., Vinh P.V., Quang N.D., Thom D.V. (2019). A refined simple first-order shear deformation theory for static bending and free vibration analysis of advanced composite plates. Materials.

[28] Anish, Chaubey A., Kumar A., Kwiatkowski B., Barnat-Hunek D., Widomski M.K. (2019). Bi-axial buckling of laminated composite plates including cutout and additional mass. Materials.

[29] Baum G.A., Brennan D.C., Habeger C.C. (1981). Orthotropic elastic constants of paper. TAPPI J..

